# The Philadelphia Electro-Therapeutic Society

**Published:** 1891-03

**Authors:** Wm. H. Walling

**Affiliations:** Secretary


### THE PHILADELPHIA ELECTRO-THERAPEUTIC SOCIETY.

WM. H. WALLING, M. D., SECRETARY.

The February meeting of this society was held at 36 North Nineteenth street, February 8. President G. Betton Massey, M. D., in the Chair. The minutes of the last meeting having been read and approved, and the Treasurer's report having been received and accepted, the society went into the election of officers for the ensuing year, with the following result:

President, Matthew W. Grier, M. D.; Vice-Presidents, I. P.

Willits, M. D.; and Horatio R. Bigelow, M. D.; Secretary and Treasurer, Wm. H. Walling, M. D.; Executive Council, Drs. G. Betton Massey, J. J. Taylor, and W. H. Walling.

DR. MASSEY then read the following paper:

ELECTRO-PUNCTURE OF A CYSTIC GOITRE; DISAPPEARANCE OF BOTH CYST AND GOITRE.

A maiden lady, aged forty-one years, was brought to me by Dr. Emily W. Wyeth, October 1, 1889, with an irregularly shaped goitre, about the size of a small orange. The left lobe was much the larger and was the seat of a monocyst of considerable proportions which had increased very much during the last year, the growth having been noticed about seventeen years. The circumference of the neck at this point was sixteen and three-eighths inches. Treatment was begun by a negative puncture of the cyst with a solid needle, 35 milliamperes being used for fifteen minutes. This was followed by a considerable oozing of a straw-colored liquid. Four days later the cyst was evacuated of its contents, measuring an ounce and a half, and 40 ma. negative applied to the cyst walls for ten minutes, by means of the canula acting as an electrode, the latter being insulated as far as the cavity. This procedure was repeated five times subsequently, with current strengths rising to 106 ma., the cavity being permitted to refill after each puncture. Careful measurements showed that the cyst was refilling more slowly after each application, but on December 9 it was decided by Dr. Wyeth and myself to make a free opening and apply the positive pole, by means of a gold bulb electrode, to all sides of the cavity at stated intervals, maintaining free drainage in the meantime. This procedure was required but twice, with currents of 100 and of 50 ma., the drainage tube, which was most assiduously looked after by Dr. Wyeth, being gradually shortened and removed on the seventh day. During this time the patient suffered a slight rise of temperature, due to a temporary obstruction of the discharge by accidental removal of the tube. By February 21, nothing remained of the growth but a cicatricial lump about the size of a peach stone, and two months later this had also disappeared without further treatment.

*Discussion.*—DR. GREER has never treated the cystic variety, but has used outward applications on true goitre, with currents of not over twenty-five ma. He used tin electrodes covered with muslin, placing the positive pole on the inferior cervical ganglion, and two negative plates upon the tumor, one on each side. The sittings lasted for five minutes each, being repeated three times a week, for from two to three months. Some preparation of the iodides was also used. Favorable results were obtained in about fifty per cent. of the cases.

DR. PETERSON spoke of a case in which the fluid extract of ergot was used, with good effect, being applied to the tumor upon the positive pole.

DR. BIGELOW : There is a canton in Switzerland in which you cannot walk out without meeting a goitre. The disease is not confined to those who drink the waters, neither to those who carry heavy burdens on their heads.

DR. BIGELOW could not see why the same treatment should not be followed in a fibroid in the neck, as well as in any other part of the body.

In a cystic tumor the action of the current was: 1, Electrolysis; 2, the arresting of the secretion; and 3, to compel absorption. He also thought that constriction should act well in such cases. Dr. Neggaroth uses the faradic current in overcoming ovarian cysts. He applies the negative pole to the ovaries, per vagina, and the positive on the abdomen, using swelling currents for an hour at a sitting, obtaining good results in six weeks. It must be the heavy voltage that acts so favorably, and if in one case, why not in another?

DR. GREER had used faradism in goitre, but abandoned it on account of its unpleasant effects.

DR. WALLING: Dr. Massey says that he emptied the cyst before applying the galvanic current. We must be guided by experience as well as by theory. Why was the positive used? Was not the negative pole the one indicated? In the treatment of hydrocele, Dr. Walling does not drain the sac, unless it is extremely distended, and then but little. Used the negative needle in the tumor, and the positive on the thigh, with a current strength of fifteen ma. for fifteen or twenty minutes. He had excellent results in such cases. Scarcely any inflammation followed, and the contents of the cysts were rapidly absorbed, with obliteration of the sacs.

He had used the strong faradic current, but saw no benefit from it, although it caused strong constractions of the muscles. Why not treat other cysts in the same way? You cannot reach all parts of the surface of the sac, after emptying it, while some parts would be unduly acted upon, tending to set up too much inflammation. What better electrolytic than the fluid in the sac, thus reaching every portion alike.

DR. MASSEY said he was disposed to regard the faradic current as of no value in cystic tumors; but in one case, where a cyst developed in a fibroid, he used a strong faradic current with great advantage. He regarded aseptic aspiration, followed by electrolytic puncture, as the best procedure in cystic conditions.

Adjourned.

---

### WHAT IS ORTHOPÆDIC SURGERY?

AT the session of the Orthopædic Section of the Tenth International Medical Congress, at Berlin, Dr. Newton M. Shaffer, of New York, read a paper in which he defined orthopædic surgery as "that department of surgery which includes the prevention, the mechanical treatment, and the operative treatment of chronic or progressive deformities, for the proper treatment of which special forms of apparatus or special mechanical dressings are necessary." While holding that the orthopædic surgeon should be a good general surgeon, yet, he says, in the field of mechanico therapy he will find abundant opportunity for the exercise of his talents without trenching on the ground of operative surgery. The wide and important field of mechanico-therapy is too often ignored in the college curriculum; and the length of treatment in these cases prevents students from taking the same interest in such patients as they take in those treated at the medical and surgical clinics. The writer makes a strong plea for the separation of the specialty from general surgery. —*The New York Medical Journal*, *February* 21, 1891.

